# Are protozoan metacaspases potential parasite killers?

**DOI:** 10.1186/1756-3305-4-26

**Published:** 2011-02-28

**Authors:** Benoît Meslin, Habib Zalila, Nicolas Fasel, Stephane Picot, Anne-Lise Bienvenu

**Affiliations:** 1 Malaria Research Unit, University Lyon 1, 8 Avenue Rockefeller, 69373 Lyon cedex 08, France; 2 Department of Biochemistry, University of Lausanne, 155 Chemin des Boveresses, 1066 Epalinges, Switzerland

## Abstract

Mechanisms concerning life or death decisions in protozoan parasites are still imperfectly understood. Comparison with higher eukaryotes has led to the hypothesis that caspase-like enzymes could be involved in death pathways. This hypothesis was reinforced by the description of caspase-related sequences in the genome of several parasites, including *Plasmodium*, *Trypanosoma *and *Leishmania*. Although several teams are working to decipher the exact role of metacaspases in protozoan parasites, partial, conflicting or negative results have been obtained with respect to the relationship between protozoan metacaspases and cell death. The aim of this paper is to review current knowledge of protozoan parasite metacaspases within a drug targeting perspective.

## Metacaspases: a twenty-first century history

In the late nineties, Aravind from Bethesda was the first to describe orthologues of caspases [1]. This paved the way for Uren and colleagues to describe paracaspases from animals and slime mould, and metacaspases from plants, fungi and protozoa, in the beginning of 21^st ^century [2]. Caspases are limited to metazoans, while metacaspases are missing from them: leading to the hypothesis that metacaspases resemble ancestral proteases and that caspases have diverged through evolution under environmental stresses. Approximately a hundred papers were published during the first decade of the new century: mostly studying the role of metacaspase in apoptosis in the budding yeast, *Saccharomyces cerevisiae*, for example [3,4]. While the involvement of yeast metacaspase in cell death is well documented [5,6], a non-death role for the yeast metacaspase Yca1p has also been described [7]. More recently, authors have strongly expressed their disagreement with the classification of metacaspases as part of the caspases family [8]. It was argued that the specificity of these enzymes should determine their classification, rather than any similarities. Other papers with definitive titles ("Are metacaspases caspases?" [9] and "Metacaspases are caspases. Doubt no more" [10]) were published, demonstrating the vitality of the debate. Whether this controversy will address issues of medical importance is debatable. However, it demonstrated the requirement to explore the role of metacaspases, as an aid to determining whether renaming these enzymes in agreement with their definitive specificity is needed.

Although only recently described and their function poorly explored, metacaspases could be considered as potential targets for new specific treatments against the main protozoan parasites affecting humans. Elucidating their role in physiology is certainly a requirement, but it is clear that addressing this issue could provide new insights into the fight against tropical diseases.

## Structure/Activity of metacaspases

Peptidases are classified in the MEROPS database http://merops.sanger.ac.uk using "clans" and "families" features. All the sequences in the same clan are evolutionarily-related, determined by similarities in protein tertiary structures and share a similar protein fold [11]. A peptidase clan consists of two letters, the first designating the catalytic type, for example A for aspartic acid, C for cysteine, S for serine. Because some cysteine, serine and threonine peptidases have similar folds, the letter "P" is used for clans of mixed catalytic type. Among the peptidases forming a transient covalent bond between the substrate and the enzyme, serine, threonine and cysteine peptidases are well described [12].

Metacaspases (MCA) are cysteine proteases with structural similarity to caspases and a catalytic cysteine-histidine dyad. Caspases and metacaspases are members of the C14 family, clan CD, with probable differences in substrate specificity [13]. Surprisingly, it was recently demonstrated that two different type 1 metacaspases from a plant (*Arabidopsis thaliana*) differentially regulate superoxide-dependant cell death: AtMC1 is a pro-death caspase-like protein while AtMC2 antagonizes this function [14]. Metacaspases have a highly acidic S1 pocket leading to a basic specificity for arginine and lysine at the P1 position, rather than aspartic acid specificity as seen for caspases [12,15].

Differences in substrate specificity between metacaspases and caspases lead to uncertainty concerning the potential role of protozoan metacaspases in apoptosis-like cell death, and it has been proposed that metacaspases and paracaspases should be separated in a specific family in clan CD [9]. Nevertheless, it was recently demonstrated that caspases and metacaspases can both target the Tudor Staphylococcal Nuclease (TSN) [16]. TSN is an evolutionary conserved protein, composed of a single Tudor domain and five staphylococcal nuclease-like domains, and is involved in a number of transcriptional and post-transcriptional pathways. *Homo sapiens *TSN sequence shows a consensus cleavage site for caspase-3, and proteolysis of TSN is blocked after treatment with the pan-caspase inhibitor zVAD-fmk. Although the DAVD caspase-3 cleavage motif is absent from protozoa, fungi and plants, TSN proteins from the plant *Picea abies *were found to contain several metacaspase cleavage sites [16]. The ability of caspase from vertebrate and type 2 metacaspase from plant to cleave the same substrate with a central role in the cell degradome provides new evidence that metacaspase could be involved in apoptosis control [16].

## *Plasmodium *Metacaspases

While insecticide-treated bednets, Artemisinin-based combined therapy (ACTs) and climatic changes have transformed the global impact of malaria in endemic areas, this parasitic disease remains a major life-threatening challenge for millions of people. ACTs are the drugs of choice for non-severe malaria, but parasite resistance is speculated to appear in the near future [17]. Thus, it is of utmost importance to decipher the mechanisms of parasite growth control, in order to detect new drug targets; potentially not subjected to resistance. Apoptosis-like DNA fragmentation of *Plasmodium falciparum *parasite in response to chloroquine treatment was described more than a decade ago [18]. It was speculated that resistance to chloroquine was related to inhibition of apoptosis. *Plasmodium berghei *features of apoptosis including condensation of chromatin, DNA fragmentation and externalisation of phosphatidylserine were demonstrated a few years later [19]. Proteins with caspase-like activity were identified in the cytoplasm of the ookinete, and more than 50% of the mosquito midgut stages of the parasite die naturally by apoptosis before gut invasion. This phenomenon was prevented by a caspase inhibitor [19].

Metacaspase, as well as clans CA, CD and CE proteases have been described in *Plasmodium *[20]. Clan CA proteases are the best-characterised cysteine proteases of *Plasmodium *(Table 1). The major functions of clan CA proteases, including falcipains, concerned with hemoglobin hydrolysis and erythrocyte rupture or invasion was extensively reviewed a few years ago [21]. However, few studies have been conducted to decipher the role of *Plasmodium *metacaspases [22,23].

**Table 1:** Key features on *Plasmodium *metacaspases

	Structure, processing and enzymatic activity	Expression, localisation and function	References
**PbMCA1**	Absence of caspase-like protease activity. C2 domain in N-terminus. P replaces the predicted catalytic C but a C is located immediately adjacent.	Expressed only in female gametocytes and all downstream mosquito stages. Absence of loss-of-function in *PbMCA1 *deficient parasites.	[23]
	
**PfMCA1**	C2 and putative CARD domains in N-terminus. Autoprocessing upstream the C14 peptidase domain. R-specific and calcium-dependant proteolytic activity. Absence of caspase-like protease activity.	Expressed in erythrocytic stages and mainly in the schizont. Induced yeast cell death and growth retardation when over-expressed in *Δyca1 *yeasts treated with H_2_O_2._	[20] [22]
	
**PfMCA2/3** **PbMCA2/3**	Absence of the H/C catalytic dyad but a C is located one residue upstream the consensus C for PfMCA3.		[22,23]

C2 domain: calcium-dependent membrane targeting motif. Abbreviations: BSF, bloodstream form; PCF, procyclic form; MCA, metacaspase; C, cysteine; D, aspartic acid; H, histidine; K, lysine; N, asparagine; P, proline; R, arginine; S, serine; W, tryptophan; FHS, fresh human serum; MLS, mitochondrial localisation signal; CARD, caspase recruitment domain.

Three MCAs were described in the genome of the two major human malaria parasites, *P. falciparum *(PfMCA1-3) and *P. vivax *(PvMCA1-3), and in the murine parasite *P. berghei *(PbMCA1-3). PxMCA1 is the only enzyme that presents the well-characterized histidine and cysteine catalytic dyad. Patterns of expression seem to differ between PfMCA1 and PbMCA1. *PfMCA1 *gene expression was described in the erythrocytic stage [Meslin et al, unpublished observations], PbMCA1 expression was detected in female gametocytes, in oocysts and in sporozoites, but authors [23] failed to demonstrate a significant level of ookinetes apoptosis compared to other studies [19,24], and did not observe differences between wild-type (WT) and PbMCA1-KO ookinetes, leading to the conclusion that functional redundancy may exist, probably through PbMCA2/3 activity. It should be kept in mind that apoptosis of normal parasites may occur in specific conditions that lead to a message of death. A more comprehensive study of the role of metacaspase in *Plasmodium *biology should be conducted in the presence of one such message. In a recent study, *Plasmodium falciparum *metacaspase expression was measured during parasite culture *in vitro*, showing a relationship with parasitemia levels and suggesting a role in growth regulation [25]. These preliminary results provide some more evidence for a role of metacaspase in *Plasmodium *fitness.

The expression of PfMCA1 C14 peptidase domain in *yca1 *deficient *S. cerevisiae *led to growth retardation and a drastic yeast cell death [Meslin et al, unpublished observations]. Interestingly, this phenotype could be blocked by the addition of the pan caspase inhibitor z-VAD-fmk while PfMCA1 did not exhibit a caspase-like, but an arginine protease activity, as reported for other protozoan MCAs [Meslin et al, unpublished observations]. PfMCA1 could play an initiator role leading to the activation of an aspartate protease effector. This hypothesis is in agreement with the description of PfMCA1 autoprocessing leading to prodomain removal as typical of initiator caspases [22]. PfMCA1 function could to be regulated by the two putative binding domains described: a C2 calcium-dependant membrane targeting domain and a CARD domain (Caspase Recruitment Domain) [22,23]. Interestingly, *Plasmodium falciparum *Tudor Staphylococcal Nuclease was recently described and some of its interacting proteins were detected using two-hybrid analysis, co-localization and co-immunoprecipitation [26,27]. *Plasmodium *Tudor SN exhibits RNA binding and nuclease activity. Whether *Plasmodium *Tudor SN could be a substrate for metacaspase is still unknown, but it probably has a central role in the parasite life cycle.

## Trypanosomatids Metacaspases

The kinetoplastid protozoan, *Trypanosoma brucei*, *Trypanosoma cruzi *and several L*eishmania *species cause, respectively, sleeping sickness, Chagas disease and various forms of leishmaniasis [28,29]. Together, these pathogenic agents affect millions of people across the world, especially in developing countries, where they are often totally mismanaged (WHO/TDR, http://www.who.int/tdr). Metacaspases have been identified and partially described in all trypanosomatids and with the recent discovery of anti-metacaspase inhibitors, further research in this area may prove to be clinically significant.

### 1 - Trypanosoma

In the African trypanosome, *T. brucei*, five genes encoding metacaspases (*TbMCA1-TbMCA5*) are expressed (Table 2). At the protein level, TbMCA2 and TbMCA3 are highly similar (89% of sequence identity), with the difference ascribed to putative organelle targeting-domain at the N-terminus of TbMCA3. TbMCA5 has a central domain, typical of a metacaspase, as well as an additional C-terminal extension [13]. Importantly, TbMCA1 and TbMCA4 have amino-acid substitutions in their conserved metacaspase catalytic residues (histidine to tyrosine and cysteine to serine in TbMCA1; cysteine to serine in TbMCA4) and are therefore expected to be enzymatically inactive [13].

**Table 2:** Key features on *Trypanosoma *metacaspases

	Structure, processing and enzymatic activity	Expression, localisation and function	References
**TbMCA1/4**	S replaces the predicted catalytic C but a C is located immediately adjacent. Absence of peptidase activity after R/D/N/K when TbMCA4 was expressed in *S. cerevisiae *or in *E. coli*.	TbMCA4 induced mitochondrial dysfunction, clonal death and growth inhibition when expressed in *S. cerevisiae*. Nuclear localisation for TbMCA4.	[13,31]
	
**TbMCA2/3/5**	No processing *in vivo *but autoprocessing for TbMCA2 after K55 and K268 in *E. coli *with calcium. Class III WW domain in N-terminus of TbMCA2/3 and in C-terminus of TbMCA5. TbMCA2 proteolytic activity: R/K-specific, Ca^2+ ^dose-dependent and processing not required. Sensitive to leupeptin, antipain, TLCK and component with R in P1 position but insensitive to E64 inhibitor.	Expression in the BSF but not in the PCF for TbMCA2/3 and in both forms for TbMCA5. No loss-of-function phenotype detected for the triple null mutants (*Δmca2/3Δmca5*). Triple RNAi induced a rapid growth arrest and a dysregulation of cytokinesis. Colocalisation of TbMCA2/3/5 with RAB11-positive endosome but no further data support involvement of TbMCAs in cell trafficking.	[30,44]
	
**TcMCA3/5**	*TcMCA3 *gene is present in 16 copies and *TcMCA5 *in a single copy in the *T. cruzi *genome. Q/P/Y rich region in the C-terminus of TcMCA5. Caspase-like activity concomitant with TcMCA3 nuclar relocalisation upon (FHS)-induced cell death.	Expression of TcMCA5 in epimastigote form. Expression of TcMCA3 in the four major stages of development. Cytoplasmic localisation and migration into the nucleus upon FHS treatment. Epimastogotes over-expressing TcMCA5 are more sensitive to (FHS)-induced cell death.	[34]

Metacaspases have also been studied in their physiological context, TbMCA2 and TbMCA3 are reportedly expressed only in the bloodstream form of the parasite whereas TbMCA5 is detectable throughout the parasite life cycle, where they appear to play a role in the recycling of endosomes co-localising with RAB11 in recycling vesicles [30]. Further, sequential genetic deletion of *TbMCA2*, *TbMCA3 *and *TbMCA5 *did not lead to cell death and even triple knockout parasites were able to recover growth rates comparable to wild-type cells. On the other hand, a rapid down-regulation of their expression using RNA-interference led to a pre-cytokinesis cell-cycle blockade; perhaps because there was inefficient time for the hypothesized compensation [13]. All five metacaspases have been studied in the heterologous organism *S. cerevisiae*, where only TbMCA4 over-expression resulted in growth inhibition and cell death. Interestingly, it was shown to be localised in the nucleus and appeared to play a role at the mitochondrial level by inducing a loss of respiratory competence in yeast cells [31].

TbMCA2 has been further biochemically characterised; it has arginine/lysine specificity at substrate P1 position [32] similar to plant metacaspases [9,33]. Despite the fact that it is auto-cleaved at lysine positions, this processing does not seem to be important for its enzymatic activity. TbMCA2 also showed a strict calcium dependency that could reach 1 mM for maximum activity; a biological concentration only encountered in acidocalcisomes. However, no experimental evidence has yet linked TbMCA2 to these compartments [32].

So far, *T. brucei *metacaspases (especially TbMCA2, TbMCA3 and TbMC5) appear to be essential in the bloodstream form of the parasite [30] and could have potential as clinically viable drug targets to fight sleeping sickness.

*T. cruzi *expresses two metacaspase proteins, one homologous to TbMCA2 (named TcMCA3) and a second homologous to TbMCA5 (named TcMCA5) [34]. The *TcMCA3 *gene is present in 16 copies per haploid genome and the encoded protein has been found to be expressed during the entire parasitic life cycle, whereas *TcMCA5 *is present as a single copy and the expressed protein is only found in the epimastigote form of the parasite. Attempts to biochemically characterize these MCAs have been unsuccessful, however Kosec *et al*. were able to show that TcMCAs could be involved in the apoptosis of the parasite as they found them to relocalise from the cytoplasm to the nucleus during apoptosis induced by fresh human serum [34]. A further caspase-like activity was reported and is most likely due to a downstream protease.

Five studies have been conducted during the last decade to explore the presence and the role of metacaspases in *Trypanosoma brucei *and *T. cruzi *parasites *in vitro*. While interesting information was obtained on similarities between *Trypanosoma *metacaspases and caspase families, proof that metacaspase activity could be similar to caspase and involved in apoptosis-like cell death is still lacking.

### 2 - Leishmania

All *Leishmania *species express one single metacaspase gene except in *infantum *and *donovani *subtypes where two metacaspases have been found encoded as GeneDB http://www.genedb.org. *Leishmania *metacaspases have a high sequence homology (77.4 to 99.8%). Structurally, they share an N-terminal domain containing a putative mitochondrial-localisation signal, a central metacaspase domain (containing the conserved catalytic dyad histidine and cysteine) and a less conserved proline-rich C-terminal domain (61.4 to 100% homology), which probably plays a role in protein-protein interactions [35] (Table 3).

**Table 3:** Key features on *Leishmania *metacaspases

	Structure, processing and enzymatic activity	Expression, localisation and function	References
**LdMC1/2**	P-rich region in C-terminus No processing with or without oxidative stress (H_2_O_2_). R/K specific proteolytic activity at pH 7.5, induced in H_2_O_2 _treated parasites. Sensitive to leupeptin, antipain and TLCK but insensitive to caspase inhibitors. Absence of caspase-like protease activity.	Expressed in both promastigote and axenic amastigote parasites. Colocalisation with the acidocalcisome compartments. Parasites over-expressing LdMCs are more sensitive to (H_2_O_2_)-induced cell death.	[36]
	
**LmjMCA**	P-rich region in C-terminus and signal peptide in N-terminus (functional MLS). Autoprocessing and R-specific proteolytic activity dependent on the H/C catalytic dyad. Absence of caspase-like protease activity. LmjMCA was extensively processed and the central catalytic domain accumulated in the cytosolic subcellular fraction upon cell death induction.	Expressed mainly in replicating amastigotes and procyclic promastigotes but less in metacyclic promastigotes. Localised in the nucleus during mitosis and in kinetoplast during organelle segregation. Promastigotes over-expressing LmjMCA suffered from growth retardation and dysregulations in kinetoplast segregation, nuclear division and cytokinesis. *Δyca1 *yeast over-expressing LmjMCA are more sensitive to (H_2_O_2_)-induced cell death. Parasites over-expressing LmjMCA showed mitochondrial over-sensitivity to H_2_O_2_.	[35,37,38]

In *L. donovani*, LdMC1 and LdMC2 were reportedly expressed in both promastigote and axenic amastigote forms of the parasite. They exhibit an arginine/lysine-specific activity without any requirement for proteolytic activation, neither in normal conditions nor upon oxidative stress-induced PCD. LdMCs have been found to be localised in the acidocalcisomes where they have been purportedly sequestered as inactive enzymes which are released upon apoptosis induction using oxygen peroxide (H_2_O_2_), as measured by the increase in metacaspase activity and TUNEL positive cells [36]. In *L. major*, the single metacaspase (LmjMCA) was found to be able to replace yeast metacaspase (YCA1) in the H_2_O_2_-induced death phenotype of *S. cerevisiae *[35]. Although it has similar activities towards arginine and lysine, in contrast to LdMCs LmjMCA was reported to be activated by autoprocessing and the purified putative catalytic domain was 300 times more active than the non-purified full-length LmjMCA [35].

The H_2_O_2_-induced death role of LmjMCA has been further characterized within the parasite. LmjMCA over-expression was found to enhance *L. major *sensitivity to oxidative stress as measured by the increase of phosphatidylserine exposure at the parasites surface and the rapid loss of mitochondrial membrane potential as compared to wild-type parasites expressing the endogenous metacaspase at physiological levels. LmjMCA was found to be extensively processed and the catalytic domain and accumulated in the cytosolic subcellular fraction when apoptosis was induced by either H_2_O_2_, heat shock or anti-*Leishmania *drugs such as miltefosine or curcumin. Even though LmjMCA has a functional N-terminal mitochondrial localisation signal, which is likely to be responsible for the partial LmjMCA mitochondrial localisation, the central catalytic domain alone was sufficient to enhance the parasite sensitivity to cell death indicating that the metacaspase would indirectly affect the mitochondrion to trigger cell death [37].

Interestingly, a non-death role has also been ascribed to LmjMCA when analyzed in its physiological context. LmjMCA was found to be expressed in actively replicating amastigotes and procyclic promastigotes, but to a lesser extent in metacyclics. It has a diffuse scattered distribution throughout the cell in interphase, becoming concentrated in the kinetoplast during organelle segregation and relocalising to the nucleus during mitosis. Here, it associates with the mitotic spindle, suggesting a role during organelle segregation and cell-cycle progression. Moreover, LmjMCA null mutants were only viable when LmjMCA was re-expressed from an episome at physiological levels, thus reinforcing its importance in parasite proliferation [38].

The discrepancies in metacaspase function and localisation range from a single metacaspase fulfilling several roles (as in *L. major*), to multiple metacaspases with structural and functional differences (as in *T. brucei*). From an evolutionary perspective, these could be of great interest as they could be used to trace the origin of the multiple roles fulfilled by the closely related caspases in metazoans.

## Targeting metacaspases to kill parasites

Caspase inhibitors are under scrutiny by several groups working on infectious diseases in humans where apoptosis promotes tissue damage. Attempts were made to understand the mechanisms involved in caspase control in human cells, in order to control pathogen-induced apoptosis. Caspase inhibitors have been tested in pre-clinical studies with various models of acute or chronic liver failure. The pan-caspase inhibitor z-VAD-fmk decreased rat mortality after massive hepatectomy [39] and IDN-6556 reduced ischemia-reperfusion injury in rat [40,41]. This latter compound was used in proof-of-concept trial with patient presenting viral hepatitis C [42] and in human liver preservation injury trial [43]. These trials, leading to inhibit human cells apoptosis, are far from the goal to induce parasite apoptosis *in vivo*. However, this shows that apoptosis control could be one of the most stimulating challenges for the future. Inhibiting human cells apoptosis to avoid tissue injury could promote cancer if the intrinsic pathway is targeted. In contrast, stimulating parasite apoptosis to increase the anti-parasitic drug efficacy could promote human cell destruction from a similar mechanism.

Considering the specificity of metacaspases activity in protozoan parasites and their absence in humans, a new research area has opened to characterize metacaspase activators or inhibitors.

Recently, a series of inhibitors have been evaluated as competitive inhibitors of TbMCA2 and 3, and on *T. brucei, T. cruzi, L. infantum *and *P. falciparum *parasites cultures *in vitro *[44]. Activities against these protozoan parasites were in the micromolar range, far from what would be needed, but the high selectivity for TbMCA2 and TbMCA3, is a first interesting step.

Whether or not protozoan metacaspases are directly involved in parasite cell death *per se*, it could be speculated that their conservation in parasites presenting a long history of adaptation to environment implies that metacaspases are key regulators of the parasite life or death decision. Thus, metacaspases should be considered as potential drug target for the future. Consideration of trials in progress using drugs designed to facilitate apoptosis of cancer cells in humans leads to speculation that similar strategies could be used to induce protozoan death at different stages of their life cycles.

Thus, it is of utmost importance to explore deeply the pathways involved in parasite apoptosis, and the key regulatory proteins that mediate cell death. In human cells, these pathways are tightly regulated at multiple steps, including through phosphorylation [45]. For example, phosphorylation of human caspase 9 Thr125 by the cyclin-dependent kinase (CDK1) - cyclin B1 inhibits caspase 9 activation. That phosphorylation is inhibited by the protein kinase inhibitor staurosporine. Additional sites of phosphorylation in caspase 9 were also reported (Ser 196, Ser 144, Ser 183, Ser195, Tyr 153, etc.) with different effects on cell apoptosis [45]. It could thus be speculated that parasite metacaspases could also be regulated by phosphorylation at specific stages of parasite cycles, and this should be taken into account to provide definitive data on the role of metacaspase in parasite death and as a drug target.

Finally, the high homology among protozoan metacaspases and the fact that these proteins are not present in mammals make them good candidates as drug targets to fight parasitic diseases. A future drug should be designed to activate the metacaspase pathway in order to favour parasite death, either directly or through the inhibition of one of the numerous negative controls suspected to be present in fast growing parasites. Inhibition of human cells apoptosis induced by parasitic diseases [46] combined with induction of parasite apoptosis at the initial steps of the infection is an integrative concept for future treatment that still needs to be proven. In both cases, recent breakthroughs in knowledge allow reasonable optimism.

## Competing interests

The authors declare that they have no competing interests.

## Authors' contributions

All authors contributed equally to the draft of the manuscript. All authors read and approved the final manuscript.
